# Deep Neural Network Quantization Framework for Effective Defense against Membership Inference Attacks

**DOI:** 10.3390/s23187722

**Published:** 2023-09-07

**Authors:** Azadeh Famili, Yingjie Lao

**Affiliations:** The Holcombe Department of Electrical and Computer Engineering, Clemson University, Clemson, SC 29634, USA; agholam@clemson.edu

**Keywords:** membership inference attack, model quantization, deep neural network, privacy, security

## Abstract

Machine learning deployment on edge devices has faced challenges such as computational costs and privacy issues. Membership inference attack (MIA) refers to the attack where the adversary aims to infer whether a data sample belongs to the training set. In other words, user data privacy might be compromised by MIA from a well-trained model. Therefore, it is vital to have defense mechanisms in place to protect training data, especially in privacy-sensitive applications such as healthcare. This paper exploits the implications of quantization on privacy leakage and proposes a novel quantization method that enhances the resistance of a neural network against MIA. Recent studies have shown that model quantization leads to resistance against membership inference attacks. Existing quantization approaches primarily prioritize performance and energy efficiency; we propose a quantization framework with the main objective of boosting the resistance against membership inference attacks. Unlike conventional quantization methods whose primary objectives are compression or increased speed, our proposed quantization aims to provide defense against MIA. We evaluate the effectiveness of our methods on various popular benchmark datasets and model architectures. All popular evaluation metrics, including precision, recall, and F1-score, show improvement when compared to the full bitwidth model. For example, for ResNet on Cifar10, our experimental results show that our algorithm can reduce the attack accuracy of MIA by 14%, the true positive rate by 37%, and F1-score of members by 39% compared to the full bitwidth network. Here, reduction in true positive rate means the attacker will not be able to identify the training dataset members, which is the main goal of the MIA.

## 1. Introduction

Machine learning is an evolving field that has recently gained significant attention and importance. With the exponential growth of data and advancements in computing power, machine learning has become a powerful tool for extracting valuable insights, making predictions, and automating complex tasks. Significant advancements in machine learning have led to the remarkable performance of neural networks in a wide range of tasks [1,2]. As the demand for real-time processing and low-latency applications continues to rise, the importance of efficient hardware implementations of machine learning algorithms becomes evident. Hardware acceleration plays a crucial role in meeting the computational requirements and enabling the deployment of machine learning models in resource-constrained environments.

To facilitate the efficient deployment of machine learning models on hardware platforms, scientists and researchers have proposed compression techniques to accelerate training and inference processes. To this end, one of the promising techniques in model compression is quantization. Quantization methods [3,4,5] accelerate the computation by executing the operations with reduced precision. These methodologies have achieved performance levels comparable to those of full bitwidth networks while remaining compatible with resource-constrained devices. These methods also enable broader possibilities for machine learning applications, particularly in sectors that handle sensitive data on the edge.

**Figure 1:**
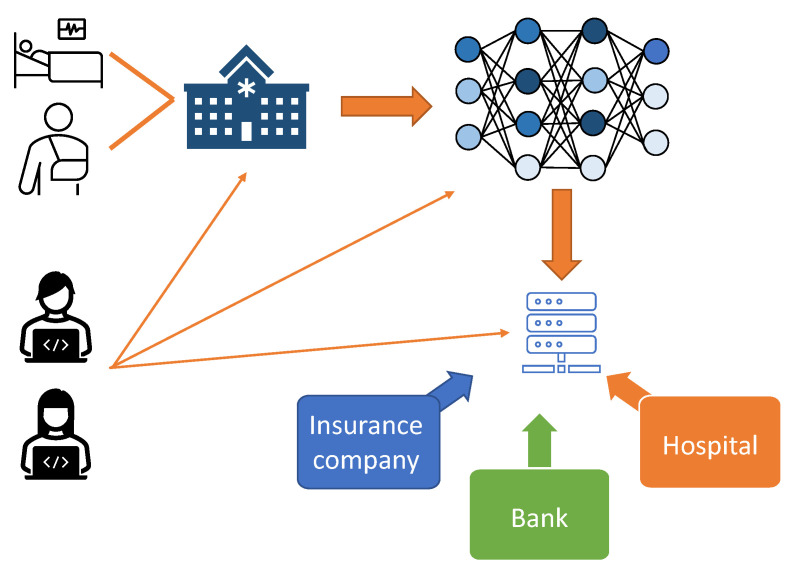
A patient medical and personal information is valuable in the field of machine learning. An adversary can jeopardize patient privacy from the machine learning models that are trained on the data.

This approach also proves valuable in various use cases, such as medical imaging [6], autonomous driving [7], facial recognition [8], and natural language processing [2], where the data privacy is of utmost importance. However, as these technologies become increasingly intertwined with daily life, they must be continuously evaluated for vulnerabilities and privacy concerns. For example, as shown in Figure 1, patient data can be used to train neural networks. In most cases, hospitals or healthcare providers gather a large amount of data regarding patients’ identity, health, insurance, and finance information. An adversary may attempt to gain access to this information at every step of this process, compromising user data privacy in machine learning applications.

Unfortunately, recent studies have demonstrated that machine learning models are quite vulnerable to well-crafted adversarial attacks [9,10,11]. For instance, adversarial attacks can easily use undetectable perturbations to deceive the models and cause misclassification. Researchers have investigated these attacks and their impact on quantized models [12,13]. It is reported in [14] that model quantization can help improve the robustness of the model against certain adversarial attacks or even be used as a defensive countermeasure. The effect of model quantization on backdoor and poisoning attacks has also been recently studied [15,16]. The extent of security and privacy risks of neural networks is not limited to adversarial attacks. On the other hand, user privacy is also of great importance to practical model deployment. In particular, membership inference attacks (MIA) can compromise the trustworthiness of a model by identifying its training dataset. MIA attack is designed to extract information regarding the training data. In a sensitive area where the training data are valuable and, in many cases, private, the MIA can lead to data leakage. Therefore, it is important to defend against MIA and limit training data leakage. To the best of our knowledge, the influence of quantized neural networks on the resistance against MIA has not been studied before.

Following the direction of this prior work, this paper proposes a novel quantization algorithm designed to enhance the resistance against MIA. The key idea is to reduce overfitting during the quantization, as MIA leverages the confidence gap between the training data and unseen data to determine the membership of a sample. To this end, our method specifically avoids overfitting and does not quantize the activations, which helps the quantized model to be more generalizable. We evaluate our method for popular model architectures on several benchmark datasets, as demonstrated in our experiment section. The quantization is performed during the backpropagation, and the algorithm uses operations such as round and clamp to constrain the weights in a predefined range. The quantization algorithm does not slow down the training phase and provides resistance to MIA.

We demonstrate that quantization not only provides increased speed but also provides resistance against MIA. Various quantization methods already exist for commercial neural network training and inference, which can facilitate the deployment of neural networks on edge devices. In practice, our method will be especially suitable for machine learning applications dealing with sensitive data before model deployment. The paper is an extended version of our previous conference paper [17]. The main contributions of this paper are summarized as follows.

- We extend our preliminary study in [17] to investigate the impact of model quantization on machine learning privacy. We demonstrate a 7 to 9 point accuracy drop in the precision of MIA attacks on quantized models compared to their corresponding full precision models.
- We propose a novel quantization algorithm where the primary goal is to enhance the resistance to MIA while also boosting efficiency.
- In our preliminary study [17], we tested the impact of quantization by using a threshold to perform MIA. In this paper, we comprehensively evaluate the proposed algorithm with a stronger form of MIA attack and training shadow models. We demonstrate that our algorithm can improve the resistance of the model to MIA in comparison to the full precision model.

The rest of the paper is organized as follows: In Section 2, we review the background of MIA and related prior work in model quantization and MIA defense. In Section 3, we discuss the MIA attack, our threat model, and our proposed quantization algorithm. Section 4 evaluates the proposed algorithm and presents the experimental results. Finally, we conclude the paper in Section 5.

## 2. Background and Related Work

### 2.1. Background

**Figure 2:**
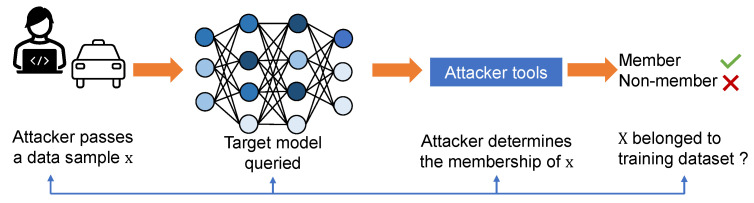
Overview of MIA attack. Here x is a data sample which the attacker wants to determine its membership.

The issue of privacy attacks in neural network training applications has raised significant concerns, particularly in sensitive scenarios [18]. Extensive research has been conducted to address the privacy implications associated with training data, focusing on various aspects such as data leakage, prevention of memorization, and evaluation of the privacy efficacy of proposed defense mechanisms. Among these, MIA has emerged as a critical concern to user data privacy in machine learning applications, as it has been shown that MIA can effectively determine whether a data sample belongs to the training set. Such MIA methods are able to extract the user data information contained in the overparameterized model. The high-level overview of MIA is shown in Figure 2. An adversary passes a data sample *x* to the target model using some analysis tools to determine the membership of this data sample.

The first MIA approach [19] uses shadow models that are trained on the same (or a similar) distribution as the training data. The method assigns membership to input and constructs a new dataset to train the classifier. Subsequently, various MIA attacks were developed considering different threat models and application scenarios. The work in [20] proves that when the adversary has data from a different but similar task, the shadow models are not needed, while a threshold reaching max prediction confidence can provide satisfactory results. The results in [21] find that the training process of ML models is the key to implementing a successful MIA. As the goal is to minimize losses associated with the training samples, members in general tend to have smaller losses than non-member samples. It has been shown that the effectiveness of MIA can be improved by using inputs from query methods [22]. The vulnerability of adversarially trained models to MIA attacks has also been exploited [23].

### 2.2. Related Work

Because we use the quantization method as a defense against MIA, we go over the state-of-the-art quantization methods and then discuss the existing defense technique against MIA.

#### 2.2.1. Model Quantization

Quantization methods have been shown to be promising in reducing the memory footprint, computational complexity, and energy consumption of neural networks. They focus on converting floating-point numbers into representations with lower bitwidth. For example, quantization can be used to reduce the model size by converting all the parameters’ precision from 32 bits to 8 bits or lower for achieving a higher compression rate or acceleration [24]. Extreme quantization is also possible where the model weights can be binary [25] or ternary [26]. In general, quantization methods can be divided into three categories.

**Traditional quantization.** In these methods, all weights and activations would be quantized. For instance, a non-uniform quantization method uses reduced bitwidth for the majority of data while a small amount of data are handled with high bitwidth [27]. A different approach in the same category utilizes a quantizer that dynamically adapts to the distribution of the parameters [28]. A quantization algorithm is developed by approximating the gradient to the quantizer step size, which can perform comparably to the full bitwidth model [29]. In [30], the proposed quantization function is a linear combination of several sigmoid functions with learnable biases and scales. The method proposed in [25] restricts weights and activations to binary values (−1,1), enabling further reduction in memory footprint and efficient hardware implementation. A more stringent quantization method uses three levels (−1,0,1) to represent weights and activations, striking a balance between binary quantization and full bitwidth.

**Mixed-precision quantization.** To avoid performance deterioration, some studies suggest using mixed-precision quantization instead of compressing all the layers to the same bidwidth. Mixed-precision quantization typically involves dividing the network into layers or blocks and applying different bitwidths to each part based on its importance and sensitivity to quantization. For example, the quantization bitwidths can be obtained by exploiting second-order (Hessian matrix) information [31]. Differentiable architecture search is also employed by [32,33] to perform mixed-precision quantization.

**Dynamic inference quantization.** Dynamic inference quantization offers several benefits, including improved flexibility, enhanced adaptability to varying run-time conditions, and potentially better accuracy than quantization with fixed bitwidth. By adjusting the quantization bitwidth on the fly, dynamic inference quantization enables efficient deployment of deep neural network models in resource-constrained environments without sacrificing accuracy. To this end, one approach is to use a bit-controller trained jointly with the given neural network for dynamic inference quantization [34]. Another study [35] proposes dynamically adjusting the quantization interval based on time step information. An algorithm developed by [36] detects sensitive regions and proposes an architecture that employs a flexible variable-speed mixed-precision convolution array.

In this paper, we develop a novel quantization method for enhancing privacy in the traditional quantization category. As the goal is to use quantization as a defense mechanism, we can ease some of the restrictions in other categories to avoid accuracy degradation. Enhancing the resistance against MIA using different quantization categories is left for future work.

**Table 1:** Prior of each table appears in numerical order. research on defense against MIA.

	Reference	Attack Knowledge	Corresponding Attack	Defense Mechanism
1	[37]	Black-box	Shadow training	Differential privacy
2	[38]	Black-box and White-box	Classifier based and Prediction loss	Distillation
3	[39]	Black-box	Classifier based and Prediction correctness	Prediction purification
4	[40]	Black-box	Shadow training	Regularization
5	[41]	Black-box	Shadow training	Regularization
6	[42]	Black-box	Classifier based	MemGuard

#### 2.2.2. Defense against MIA

A defense mechanism against MIA, named MemGuard, was developed [42], which can evade the attacker’s membership classification and transform the prediction scores into an adversarial example. MemGuard adds a carefully crafted noise vector to the prediction vector and turns it into an adversarial example of the attack model. Differential privacy [43,44], which can provide a probabilistic guarantee of privacy, has also been shown to be effective in enhancing resistance against MIA [37]. However, differential privacy is costly to implement, and the accuracy reduction makes the method impractical. Distillation for membership privacy (DMP) is a method proposed by [38]. DMP first trains a teacher model and uses it to label data records in the unlabeled reference dataset. The teacher method has no defense mechanism. DMP requires a private training dataset and an unlabeled reference dataset. The purifier framework [39], where the confidence scores of the target model are used as input and are purified by reducing the redundant information in the prediction score, has also been proposed to defend against MIA.

On the other hand, regularization methods designed to reduce overfitting in machine learning models can be employed as defense strategies against MIAs. Adversarial regularization [40] and Mixup + MMD [41] are specific regularization techniques intended to mitigate MIAs. Using regularization, the model generalization is improved and the gap between member and non-member data samples is reduced. However, the privacy risks after implementing these methods are still high [45]. In Table 1, we summarized prior work based on attack knowledge, MIA attack, and defense mechanism. To the best of our knowledge, using quantization to enhance the resistance against MIA has not been investigated before.

## 3. Proposed Defense to MIA

We found quantization could help improve the resistance against MIA in our prior work [17]. Our results showed that quantized models would have a lower MIA attack accuracy compared to the corresponding full bitwidth models. We also demonstrated that using a quantization method can reduce the precision of the attack while recall stays similar. The results are even more pronounced when we deal with more complicated tasks. We showed that the F1-score of MIA can be reduced by 7% after quantization. Built upon our prior findings, in this section we propose a novel quantization method that can further improve the resistance against MIA.

### 3.1. Threat Model

In membership inference attacks given sample data *x*, an adversary tries to infer whether *x* is a member of the training dataset. We consider a threat model that is consistent with prior work on MIA [21,46,47,48]:

- **Access the target model**: We assume the adversary could only access the target model output. This is referred to as black-box access [41].
- **Access to the data**: Although the adversary does not have access to the training data, we assume the adversary can sample from the available pool of data that has the same distribution as the training data.

The model will be quantized using our proposed method after training. The adversary then might perform MIA against the quantized model.

### 3.2. MIA Algorithm

All the symbols and their definitions are summarized in Table 2. Conventionally, to perform an MIA, the adversary has access to a dataset $D_{s}$ with similar distribution to the target model training dataset $D_{t}$. Using the dataset $D_{s}$, the adversary trains their shadow model $f_{s}$ in a way that the shadow model has a similar behavior as $f_{t}$. The adversary then uses the shadow model’s confidence vector to train a binary classifier $f_{a}$, which typically is a multi-layer perception (MLP). This model $f_{a}$ is trained on the confidence vectors and label∈(0,1), where 0 and 1 represent non-member and member, respectively. To determine the membership, the data sample *x* is given to the shadow model $f_{s}$, then the confidence vector is given to the attack model $f_{a}$.

An alternate way to implement the MIA attack is to use the confidence vector of the $f_{t}$ and predict highly confidant samples *x* as members using confidence thresholding T. We used this method in our prior work [17]. However, this method focuses on the confidence vector of the target model $f_{t}$. As discussed in Section 2.2.2, several prior defensive methods have shown significant resistance against this type of attack. Thus, we use the shadow model method to predict the membership of sample *x* in this paper.

### 3.3. Proposed Quantization Scheme

**Figure 3:**
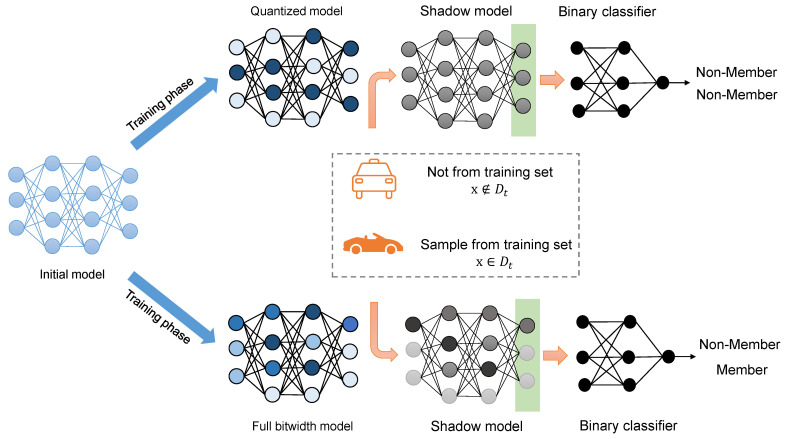
Overview of the quantization and the MIA attack setup. The top represents the quantized model, and the bottom represents the full bitwidth model. The shadow model is trained to imitate the target model. The final layer of the shadow model provides the confidence vector, which is then used to train a binary classifier that provides the final membership decision. The goal is to leverage quantization to enhance the resistance against MIA. For example, a training sample will be identified by MIA on the full bitwidth model, whereas it might not be recognized as a member after quantization.

To deploy neural network models on edge devices, model quantization is used to replace floating point values with lower bitwidth representations. Our setting is shown in Figure 3. The key of our proposed method is to reduce overfitting during the quantization, as MIA leverages the confidence gap between the training data and unseen data to determine the membership of a sample. To this end, our method specifically avoids overfitting and does not quantize the activations, which helps the quantized model to be more generalizable.

A neural network model is denoted as f(x;W), consisting of *n* layers represented by $L_{1},L_{2},\cdots ,L_{n}$. We can perform quantization on the weights $W=W_{1},\cdots ,W_{n}$, where each layer $L_{i}$ has a set of weights $W_{i}$. The quantization function can be defined as follows:

(1) $$Q\left(w_{i}\right)=\gamma_{j}\;\forall w_{i}\in \left(p_{j},p_{j+1}\right]$$

In Equation (1), $\left(p_{j},p_{j+1}\right]$ represents a real number interval, where *j* ranges from 1 to $2^{b}$, and *b* corresponds to the quantization bitwidth. The values of $w_{i}$ to be quantized can be tensors with floating-point values. The quantization function maps all $w_{i}$ values within the defined range to a specific quantized value $\gamma_{j}$.

Conventionally, researchers often employ a unified quantization function that divides the range equally into intervals. This approach ensures that the step size, denoted as *s*, is calculated as the range of values divided by the number of intervals. Mathematically, the quantization process can be expressed as:

(2) $$W_{r}=R\left(\frac{w}{s}\right),$$

(3) $$s.t.\;s=\frac{r_{1}-r_{0}}{2^{b}}$$

where R represents the rounding function, which rounds the result of $\frac{w}{s}$ to the nearest integer. The initial range $\left(r_{0},r_{1}\right)$ is divided into $2^{b}$ intervals. In quantization, *s* and *b* can significantly impact the training. We chose the interval by clamping the $r_{1}$ to $2^{b}-1$; the final step can be written as:

(4) $$W_{Q}=min\left(max\left(W_{r},r_{0}\right),r_{1}\right).$$

**Figure 4:**
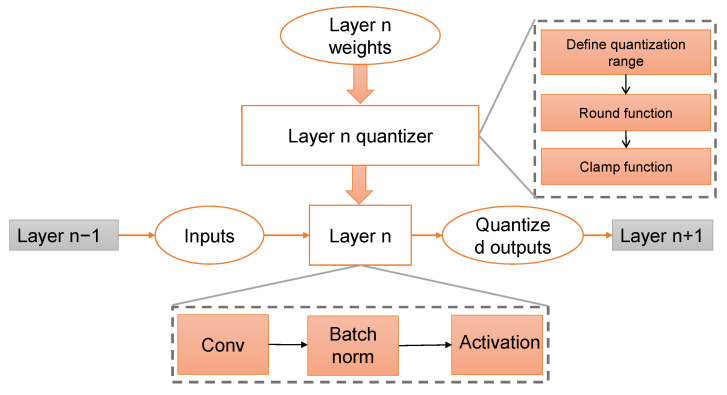
Overview of the proposed methodology. Each layer’s weight is passed through the quantizer. After defining the quantization range, the parameters are passed through two functions (round and clamp).

The layerwise operation of the proposed method is shown in Figure 4. During the training of the model f(x;W), the weights $W_{L_{i}}$ of each layer $L_{i}$ get updated. To ensure a smooth training process, we apply quantization to each $W_{L_{i}}$ in a manner that does not disrupt the training. It is crucial to avoid any disturbance to the training process because doing so would require retraining the model with the same dataset, increasing the risk of overfitting and eventually MIA. We present the process flow in Algorithm 1.

Our algorithm is a uniform weight-only quantization. Given the model f(x,W), at each stage of training *t*, we perform quantization for each input. We find $r_{1}$, which is referred to as nbins in Algorithm 1. In the next step, we calculate $W_{C}^{L_{i}}$, where *C* stands for clamped weights, which is the operation described in Equation (4). Using zero point *z*, we can offset the range. We use zero point z=2 for our experiment, as we find it empirically works well against MIA.
**Algorithm 1** The training procedure of the proposed quantization scheme.**Require:** Original DNN parameterized f(x;W), *b*, *s*, *z*, *L*
 Set epochs *T* for training **for**
*T*
**do**    nbins=pow(2,nbits)+1    **for** *i* in *L* **do**
    $W_{C}^{L_{i}}=clamp\left(R\left(W_{L_{i}}/s\right)+z,min=0,max=nbins\right)$    $W_{Q}^{L_{i}}=s*\left(W_{C}^{L_{i}}-z\right)$▹ After backpropagation weights for each layer are quantized    **end for** **end for**

## 4. Experimental Results

### 4.1. Experimental Settings

For evaluation, we use the widely used datasets, neural network architectures, and optimization approaches following recent work in MIA [21,23]. The experimental settings, including the selections of model architectures and datasets, are consistent with prior work on MIA [19,21,23]. We compare our method to full bitwidth networks, whose weights are represented in 32-bit floating point values.

#### 4.1.1. Datasets

**Fashion MNIST** [49]. Fashion MNIST consists of a training set of 60,000 images. Each image is a 28 × 28 grayscale, with labels from 10 classes. The dataset has 10,000 images for testing. We applied several data augmentation techniques, including random cropping and random rotation, for the training process.

**Cifar10** [50]. Cifar10 is a widely used benchmark dataset for image classification. The Cifar10 dataset consists of 60,000 color images with dimensions of 32 × 32 pixels distributed across 10 distinct classes. Each class contains 6000 images.

#### 4.1.2. Model Architectures

**ResNet**: We use the ResNet architecture [51] to train the target and shadow models on Cifar10. We perform our experiments in both ResNet-20 and ResNet-50; the numbers refer to the depth of the ResNet architecture. We use an MLP binary classifier with one hidden layer for the attack model.

**LeNet**: For Fashion MNIST, we use LeNet [52] to train the target and shadow models.

#### 4.1.3. MIA Algorithms

As discussed in Section 2.1, we perform the attack using a shadow model. We use 15,000 samples of the dataset (Cifar10 or Fashion MNIST) to train the $f_{target}$, and another 15,000 samples to train the shadow model $f_{shadow}$. Two sets of 15,000 samples are used for testing in the MIA attack. We use the trained shadow model to make the training feature dataset for the MLP.

#### 4.1.4. Baseline Quantization Method

We utilize the method developed in [4], DoReFa-Net, for baseline comparison. Although DoReFa-Net was tested only on AlexNet in the original paper, it has excellent performance on ResNet. The method first limits the values of the weights to [−1,1] and then quantizes them to the desired bitwidth within the range [0,1]. DoReFa-Net quantizes both weights and activations.

### 4.2. Results

As discussed in [53], it is essential to evaluate MIA results comprehensively to show the effectiveness of the method. The model’s accuracy does not provide enough insights to judge the effectiveness of the attack or defense performance. We report the target model accuracy, shadow model accuracy, and attack model accuracy in Table 3. We report each class’s precision, recall, and F1-score in Table 4. To have more insight into the performance of the proposed algorithm, we present attack accuracy, true positive, true negative, false positive, and false negative rate in Table 5. We train the target model for 50 epochs and divide the data between training the target model and the shadow model. The results in Table 3, Table 4 and Table 5 are reported when the target model is quantized, but the shadow model is trained with full bitwidths.

As shown in Table 3, we quantize our algorithm with two different bitwidths of 4 and 8. In the case of ResNet-50, the quantized model has a better target model accuracy than the full bitwidth model. However, the attack accuracy drops when compared to the quantized model. As we can see from Table 3, the shadow model of the full bitwidth model has higher accuracy than its quantized counterpart on Fashion MNIST, whereas that is not the case for Cifar10. When the shadow model is being trained, the goal is to imitate the behavior of the target model, instead of achieving the best accuracy. In our experiments, the shadow model fails to learn the behavior of the quantized model, but it achieves a higher accuracy for the Cifar10 data. In contrast, the shadow model learns the behavior of the full bitwidth model, and the attack model accuracy demonstrates the effectiveness of the attack. We also find that the attack model accuracy is lower on Fashion MNIST than on Cifar10. However, the shadow model accuracy is higher for Cifar10. We test three quantized networks with bitwidths of 4, 8, and 16. Out of all quantized networks, 16-bit quantization behaves similarly to the full bitwidth model. However, the attack model accuracy is still lower than for the full bitwidth model.

In Table 4, we present more evaluation metrics, including precision, recall, and F1-score for both member and non-member classes. Here, recall refers to:

(5) $$Recall=\frac{TP}{TP+FN}.$$

Precision is defined as:

(6) $$Precision=\frac{TP}{TP+FP}.$$

Finally, F1-score refers to:

(7) $$F1\text{-}score=\frac{2*Precision*Recall}{Precision+Recall}.$$

We can see a significant drop in all three metrics when we compare the member class of the ResNet-50 full bitwidth model to the quantized models. The same trend can also be observed for ResNet-20 and the simpler LeNet model.

We evaluate true negative (TN), false positive (FP), false negative (FN), and true positive (TP) to show the effectiveness of our method in Table 5. Here, true negative refers to instances where non-members are correctly identified as non-members. A false positive occurs when non-members are incorrectly identified as members. A false negative happens when members are mistakenly identified as non-members. Finally, true positive denotes instances where members are correctly identified as members.

It can be seen that the true positive rates are lower after employing our quantization, which shows the effectiveness of our proposed scheme. Furthermore, although we observe only a small difference in attack accuracy for the 8-bit quantized network on ResNet-20, compared to the full bitwidth network, there is a nearly 30-point reduction in TP value. This means that the attacker can only determine non-members, and it is falsely classifying members of training sets as non-members. This can also be indicated from the FN of the 8-bit quantized network, which shows the effectiveness of our method.

**Figure 5:**
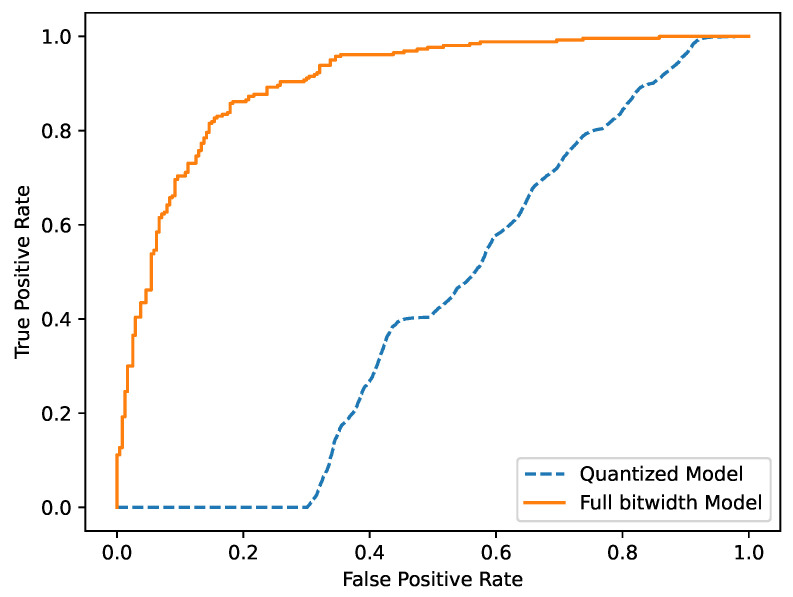
ROC of quantized ResNet-20 and the corresponding full bitwidth model.

In addition, we provide the ROC curve of MIA, as shown in Figure 5. It can be seen that the MIA on the full bitwidth model is successful while behaving as a random classification on the quantized model.

Finally, we compare our algorithm to DoReFa-Net [4] in Table 6. We can see the impact of MIA is significantly reduced in both quantization methods. Compared to DoReFa-Net, our method achieves further reductions in all three metrics, verifying the advantages of our method. For instance, our method achieves over 28% reduction in F1-score compared to DoReFa-Net for the 4-bit quantization. We can see the DoReFa-Net reduces the effectiveness of the MIA. However, compared to DoReFa-Net, our method achieves further reductions in all three metrics, verifying the advantages of our method. For instance, our method achieves over 28% reduction in F1-score compared to DoReFa-Net for the 4-bit quantization.

**Figure 6:**
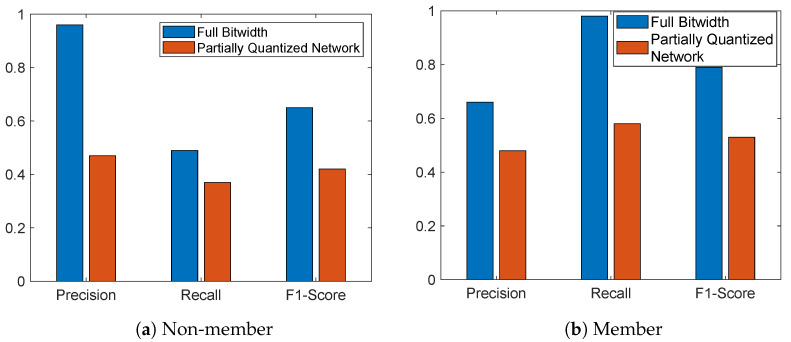
The effectiveness of MIA, when ResNet-20 is partially quantized in comparison to the full bitwidth model.

We also conducted an ablation study to determine if our method can still provide protection against MIA when we only partially quantize the model network. We present the results on ResNet-20 in Figure 6. This network has 42 layers with weights that can be quantized. In this experiment, we only quantize the last 5 layers to 4-bit. It can be seen that in both members and non-members, the partially quantized network has lower F1-score, precision, and recall. We can see a significant drop in Precision for the non-member class, which also leads to greatly reduced TP values in the partially quantized model.

## 5. Conclusions

This paper presented a novel quantization method for defending against MIA. Quantization has been shown to be effective in model compression and efficiency improvement. We demonstrated that quantization techniques can also be used as a countermeasure against user data privacy leakage in neural networks. We showed that our proposed algorithm could specifically reduce the effectiveness of MIA by lowering the true positive and increasing the false negative rate.

## Figures and Tables

**Table 2:** The notations used in the paper.

Symbol	Definition
$f_{s}$	Shadow model
$f_{t}$	Target model
$f_{a}$	Attack model (binary classifier)
$D_{s}$	Shadow model dataset
$D_{t}$	Target training dataset

**Table 3:** The accuracy of the shadow and attack models.

Model	Bitwidth	Shadow Model Accuracy	Attack Model Accuracy
LeNet	4	82.39%	50.07%
	8	83.20%	50.21%
	16	83.02%	50.20%
	full	88.26%	53.40%
ResNet-20	4	51.22%	69.50%
	8	51.38%	72.50%
	16	70.62%	66.87%
	full	60.58%	72.30%
ResNet-50	4	58.90%	64.10%
	8	60.38%	59.38%
	16	67.70%	56.89%
	full	54.01%	71.10%

**Table 4:** F1-score, precision, and recall of the full bitwidth model and quantized model.

Model	Bitwidth	Class	*Precision*	*Recall*	*F*1-*Score*
LeNet	4	Non-Member	0.51	0.06	0.11
		Member	0.50	0.94	0.65
	8	Non-Member	0.51	0.16	0.24
		Member	0.50	0.85	0.63
	16	Non-Member	0.50	0.23	0.31
		Member	0.50	0.78	0.61
	full	Non-Member	0.64	0.23	0.34
		Member	0.53	0.87	0.66
ResNet-20	4	Non-Member	0.52	0.82	0.64
		Member	0.58	0.26	0.36
	8	Non-Member	0.57	0.73	0.64
		Member	0.62	0.44	0.52
	16	Non-Member	0.76	0.49	0.60
		Member	0.62	0.85	0.72
	full	Non-Member	1.00	0.35	0.52
		Member	0.61	1.00	0.75
ResNet-50	4	Non-Member	0.59	0.50	0.54
		Member	0.57	0.65	0.61
	8	Non-Member	0.65	0.41	0.50
		Member	0.57	0.78	0.66
	16	Non-Member	0.56	0.60	0.58
		Member	0.57	0.54	0.55
	full	Non-Member	0.95	0.37	0.54
		Member	0.61	0.98	0.75

**Table 5:** Attack accuracy, TN, FP, FN, and TP for full bitwidth and quantized networks.

Model	Bitwidth	Attack Accuracy	*TN*	*FP*	*FN*	*TP*
LeNet	4	50.07%	03.24%	46.76%	3.17%	46.82%
	8	50.21%	07.79%	42.21%	7.57%	42.42%
	16	50.20%	11.29%	38.70%	11.09%	38.90%
	full	54.89%	11.50%	38.50%	6.61%	43.39%
ResNet-20	4	53.59%	40.76%	9.23%	37.18%	12.82%
	8	65.88%	36.30%	13.69%	27.86%	22.13%
	16	66.84%	23.50%	26.49%	06.66%	43.33%
	full	67.54%	17.56%	32.43%	0.02%	49.97%
ResNet-50	4	57.66%	25.09%	24.90%	17.43%	32.56%
	8	59.38%	20.47%	29.52%	11.09%	38.90%
	16	56.89%	30.13%	19.86%	23.24%	26.75%
	full	67.69%	18.68%	31.32%	1.02%	49.00%

**Table 6:** Performance comparison of DoReFa-Net and our quantization algorithm MIA on ResNet-20.

Method	Bitwidth	*F*1-*Score*	*Precision*	*Recall*
DoReFa-Net	4	70.12	54.00	100.00
	16	71.79	56.00	100.00
Proposed	4	50.00	55.00	64.00
	8	58.00	59.05	59.50
	16	66.00	88.05	67.00
	full	77.30	63.00	100.00

## Data Availability

Not applicable.
